# Lv4 Is a Capsid-Specific Antiviral Activity in Human Blood Cells That Restricts Viruses of the SIV_MAC_/SIV_SM_/HIV-2 Lineage Prior to Integration

**DOI:** 10.1371/journal.ppat.1005050

**Published:** 2015-07-16

**Authors:** Massimo Pizzato, Sean Matthew McCauley, Martha R. Neagu, Thomas Pertel, Claudia Firrito, Serena Ziglio, Ann Dauphin, Madeleine Zufferey, Lionel Berthoux, Jeremy Luban

**Affiliations:** 1 Department of Microbiology and Molecular Medicine, University of Geneva, Geneva, Switzerland; 2 Center for Integrative Biology, University of Trento, Trento, Italy; 3 Program in Molecular Medicine, University of Massachusetts Medical School, Worcester, Massachusetts, United States of America; 4 Laboratory of Retrovirology, University of Québec, Trois-Rivières, Quebec, Canada; Fred Hutchinson Cancer Research Center, UNITED STATES

## Abstract

HIV-2 and SIV_MAC_ are AIDS-causing, zoonotic lentiviruses that jumped to humans and rhesus macaques, respectively, from SIV_SM_-bearing sooty mangabey monkeys. Cross-species transmission events such as these sometimes necessitate virus adaptation to species-specific, host restriction factors such as TRIM5. Here, a new human restriction activity is described that blocks viruses of the SIV_SM_/SIV_MAC_/HIV-2 lineage. Human T, B, and myeloid cell lines, peripheral blood mononuclear cells and dendritic cells were 4 to >100-fold less transducible by VSV G-pseudotyped SIV_MAC_, HIV-2, or SIV_SM_ than by HIV-1. In contrast, transduction of six epithelial cell lines was equivalent to that by HIV-1. Substitution of HIV-1 CA with the SIV_MAC_ or HIV-2 CA was sufficient to reduce HIV-1 transduction to the level of the respective vectors. Among such CA chimeras there was a general trend such that CAs from epidemic HIV-2 Group A and B isolates were the most infectious on human T cells, CA from a 1° sooty mangabey isolate was the least infectious, and non-epidemic HIV-2 Group D, E, F, and G CAs were in the middle. The CA-specific decrease in infectivity was observed with either HIV-1, HIV-2, ecotropic MLV, or ALV Env pseudotypes, indicating that it was independent of the virus entry pathway. As_2_O_3_, a drug that suppresses TRIM5-mediated restriction, increased human blood cell transduction by SIV_MAC_ but not by HIV-1. Nonetheless, elimination of TRIM5 restriction activity did not rescue SIV_MAC_ transduction. Also, in contrast to TRIM5-mediated restriction, the SIV_MAC_ CA-specific block occurred after completion of reverse transcription and the formation of 2-LTR circles, but before establishment of the provirus. Transduction efficiency in heterokaryons generated by fusing epithelial cells with T cells resembled that in the T cells, indicative of a dominant-acting SIV_MAC_ restriction activity in the latter. These results suggest that the nucleus of human blood cells possesses a restriction factor specific for the CA of HIV-2/SIV_MAC_/SIV_SM_ and that cross-species transmission of SIV_SM_ to human T cells necessitated adaptation of HIV-2 to this putative restriction factor.

## Author Summary

HIV-1 and HIV-2, the two lentiviruses that cause AIDS in humans, are members of a family of such viruses that infect African primates. HIV-1 is a zoonosis that was transmitted to humans from chimpanzees. HIV-2 was transmitted to humans from sooty mangabey monkeys. In several documented cases of cross-species transmission of lentiviruses it has been shown that replication of the virus in the new host species necessitated that the virus adapt to species-specific antiviral factors in the host. Here we report that human blood cells possess an antiviral activity that exhibits specificity for viruses of the HIV-2/SIV_MAC_/SIV_SM_ lineage, with restriction being greatest for SIV_SM_ and the least for epidemic HIV-2. Here we show that this dominant-acting, antiviral activity is specific for the capsid and blocks the virus after it enters the nucleus. The evidence suggests that, in order to jump from sooty mangabey monkeys to humans, the capsid of these viruses changed in order to adapt to this antiviral activity. In keeping with the practice concerning anti-lentiviral activities we propose to call this new antiviral activity Lv4.

## Introduction

Human immunodeficiency virus type 1 (HIV-1) is the major cause of the acquired immune deficiency syndrome (AIDS) pandemic. Among the immunodeficiency viruses that infect at least 40 of the primate species in sub-Saharan Africa, the simian immunodeficiency viruses (SIVs) found in central African chimpanzees and gorillas are monophyletic with HIV-1 [1,2]. Each of the four HIV-1 lineages (groups M, N, O, and P) is believed to have resulted from independent cross-species transmission of simian immunodeficiency viruses from chimpanzees (SIV_CPZ_), and perhaps from gorillas (SIV_GOR_) [3–6]. SIV_CPZ_ itself is probably a recombinant virus that resulted from co-infection of a chimp with viruses transmitted from a red-capped mangabey (SIV_RCM_) and a greater spot-nosed monkey (SIV_GSN_) [7]. Until recently it was believed that SIV_CPZ_ did not cause disease in chimpanzees but extensive observation of feral animals has demonstrated that this is not the case [8].

HIV-2, a second AIDS-causing virus that has highest prevalence in West Africa, was transmitted to people from sooty mangabey monkeys (*Cercocebus atys*) on multiple occasions [9–12]. There is no evidence for disease in sooty mangabey monkeys infected with SIV_SM_, but cross-species transmission to another non-native host, rhesus macaques (SIV_MAC_), resulted in AIDS [13,14].

Though transmission of primate lentiviruses to humans has occurred on multiple occasions and may still be occurring [15], these events are probably uncommon. Primate lentiviral sequences can be grouped into clades that are specific for a given host species [2]. Species crossovers are prevented in part by innate immune mechanisms, of which restriction by intracellular proteins is an important component. Proteins of the TRIM (Tripartite Motif) family can disrupt retroviral replication in a species-dependent manner [16–18]. TRIM proteins displaying anti-retroviral activity are present in all primates tested so far [19]. Moreover, phylogenetically and functionally related genes have been found in cattle [20,21] and in rabbits [22]. TRIM5α was the first member of this family to be identified as an anti-retroviral gene [23] and has been extensively studied. It targets incoming susceptible retroviruses, trapping them in cytoplasmic bodies that seem to form around the virus [24]. Inhibition of retroviral replication requires specific recognition of retroviral capsid motifs, and a TRIM5α-CA interaction can be detected in various settings [25–27]. Additionally, treatment with proteasome inhibitors partially relieves the restriction, suggesting that TRIM5α targets susceptible retroviruses to a proteasomal degradation pathway [28–30]. Finally, TRIM5α prevents nuclear transport of restricted retroviruses [28,30–32].

HIV-1 is inhibited by TRIM5α from a number of African and Asian monkey species, such as rhesus macaques, African green monkeys, and sooty mangabeys [19,33]. The human orthologue of TRIM5α restricts some non-primate lentiviruses such as the N-tropic strains of the murine leukemia virus (N-MLV) and the equine infectious anemia virus (EIAV) [34–36]. However, it has minimal activity against HIV-1 and various strains of SIVs such as SIV_MAC_ and SIV from African green monkeys (SIV_AGM_) [32,36–39].

Thus, available data suggest that the early post-entry stages of SIV_MAC_ replication are not inhibited by TRIM5α in human cells. These experiments, however, all used immortalized adherent cell lines such as TE671 (rhabdomyosarcoma) [32,40,41], HOS (osteosarcoma) [42] or HeLa cells (adenocarcinoma) [23,31,43]. Hofmann and colleagues compared the infectivity of vectors derived from SIV_MAC_ or HIV-1 in a range of mammalian cell lines [44]. They found that HIV-1 vectors were up to 9-fold more infectious than SIV_MAC_ vectors in several human cell lines, e.g. Raji (B lymphocyte) and in the T lymphocyte cell lines Jurkat, HuT78 and CEM. This raised the possibility that lentiviruses could be inhibited in a cell-type specific fashion in human cells. In the work presented here, we investigated restriction to SIV_MAC_ replication in peripheral blood lymphocytes (PBLs) as well as in various cell lines. Our data reveal a TRIM5α-independent restriction activity targeting SIV_MAC_, and the related SIV_SM_ and HIV-2, in human blood cells.

## Results

### Human blood cells are less permissive for SIV_MAC_, SIV_SM_, and HIV-2, than for HIV-1

**Fig 1:**
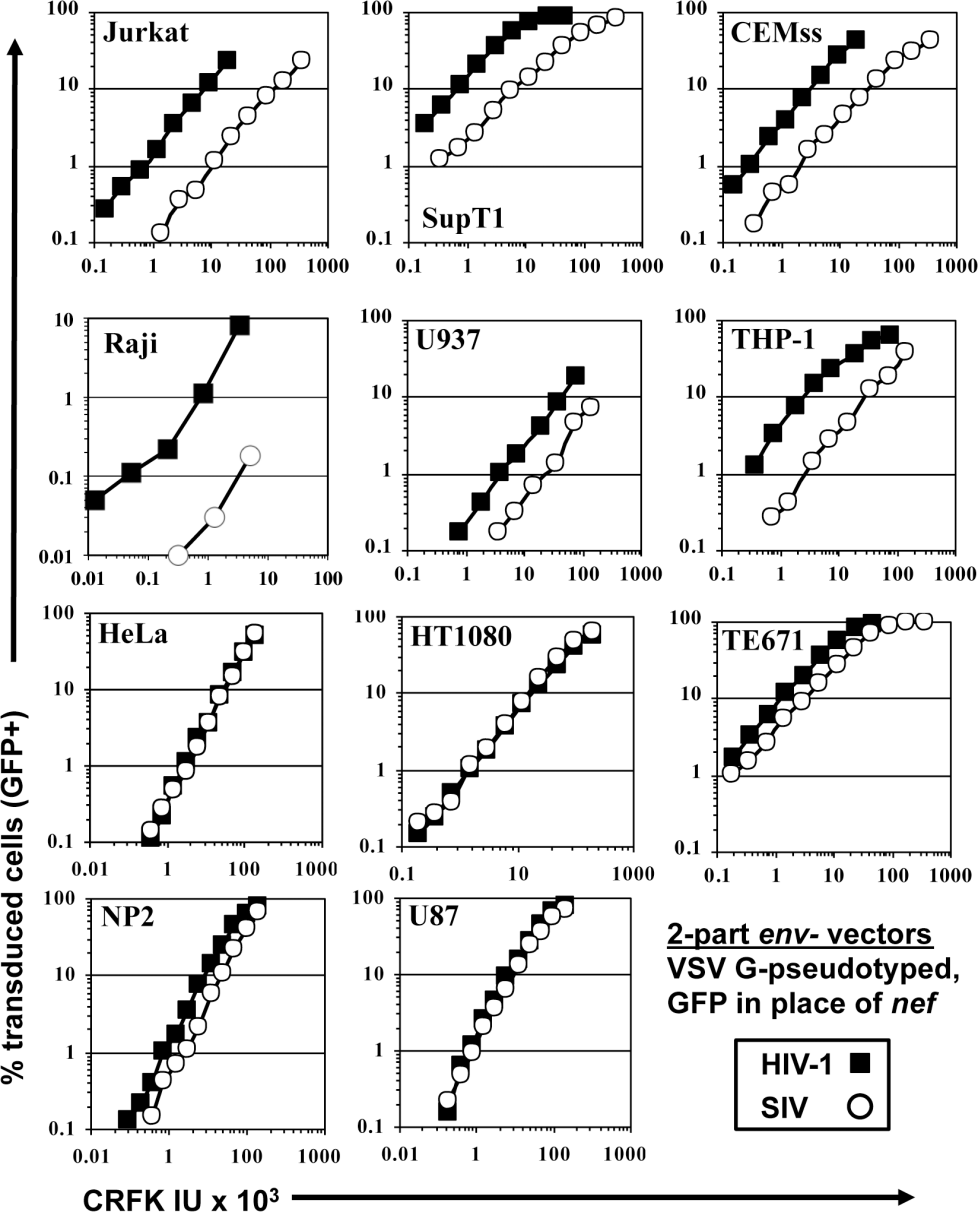
SIV_MAC_ transduction of human blood-derived cell lines is less efficient than is transduction by HIV-1. VSV G-pseudotyped HIV-1_NL4-3_GFP (black squares) and SIV_MAC_239GFP (white circles) were generated by plasmid transfection of 293T cells. In each plasmid, *env* was disrupted and *nef* replaced with GFP, such that the fluorescent reporter gene was expressed from the 5’ LTR. Vector stocks were normalized by titer on CRFK cells, and then used to challenge the indicated cell lines. 48 hrs post vector challenge, the percentage GFP-expressing cells was determined by FACS. Data is plotted as percent GFP^+^ (infected) cells (Y axis) versus CRFK infectious units (IU) x 1,000 (X axis).

Human cell lines were challenged with VSV G-pseudotyped, single-cycle vectors derived from HIV-1_NL4-3_ and SIV_MAC_239, as previously described [45]. In each case, *nef* was replaced with GFP coding sequence, such that the fluorescent reporter was expressed from the respective LTR. The two vectors were produced in parallel by collecting supernatant from transfected 293T cells. The vector-containing supernatants were checked for reverse transcriptase activity [46], normalized for titer on highly permissive CRFK feline kidney epithelial cells [47], and then used to infect a panel of human cell lines by serial dilution (Fig 1).

SIV_MAC_ transduction efficiency was 4 to 20-times less than that of HIV-1_NL4-3_ when the two vectors were used to challenge any of a panel of T cell lines, including Jurkat, SupT1, and CEM-SS cells, the Burkitt lymphoma-derived B cell line Raji, or the myelomonocytic cell lines U937 and THP-1 (Fig 1). The infectivity of SIV_MAC_ was similar to that of HIV-1_NL4-3_ in adherent epithelial cell lines, including HeLa cells, HT1080 fibrosarcoma cells, TE671 rhabdomyosarcoma cells, U87 glioblastoma cells, and NP2 glioma cells (Fig 1).

**Fig 2:**
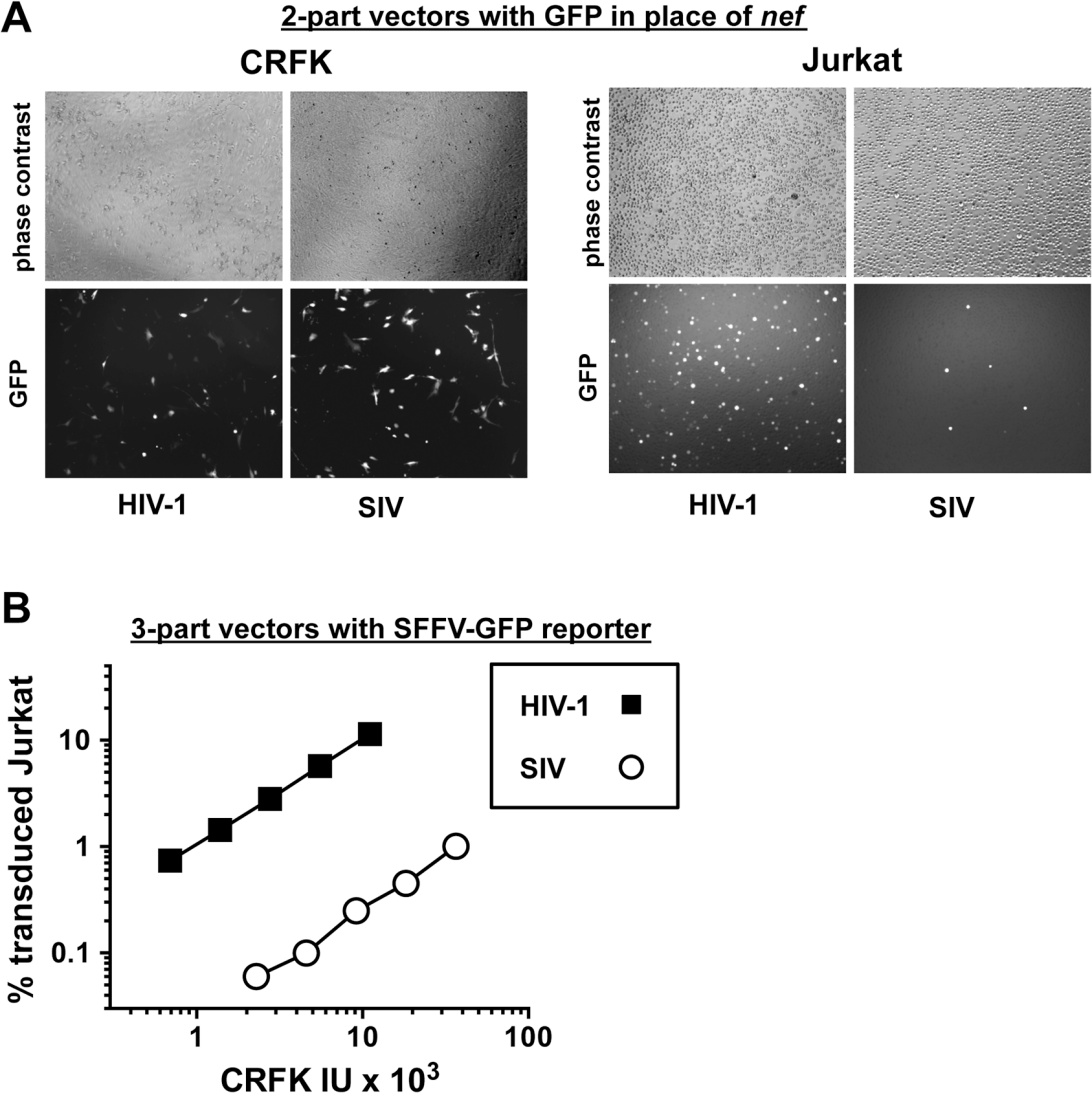
The decrease in T cell transduction efficiency by SIV_MAC_ is not explained by differences in reporter gene expression. (A) CRFK cells (left panel) and Jurkat T cells (right panel) were transduced with VSV G-pseudotyped, single-cycle, two-part HIV-1_NL4-3_GFP or SIV_MAC_239-GFP vectors, as in Fig 1_._ Virus stocks were normalized by reverse transcriptase activity prior to transduction. 48 hrs after transduction, cells were visualized by phase contrast and fluorescence microscopy. Shown are representative fields for each condition at 100x magnification. Fluorescence intensity of individual T cells transduced with SIV_MAC_239-GFP is at least as strong as that in cells transduced with HIV-1_NL4-3_GFP. (B) VSV G-pseudotyped, HIV-1_NL4-3_ (black squares) and SIV_MAC_239 (white circles) three-part vectors were generated by plasmid transfection of 293T cells. In each case, the viral genomic RNA was designed to transduce an identical SFFV-GFP reporter gene. Vector stocks were normalized by titer on CRFK cells, and then used to challenge Jurkat T cells. 48 hrs post vector challenge, the percentage GFP-expressing cells was determined by FACS. Data is plotted as percent GFP^+^ (infected) cells (Y axis) versus CRFK infectious units (IU) x 1,000 (X axis).

Signal intensity by immunofluorescence microscopy of individual GFP-positive cells after SIV_MAC_ transduction was at least as great as that after HIV-1_NL4-3_ transduction (Fig 2A). Mean fluorescence intensity by flow cytometry was 219.6 +/- 15.5 for SIV_MAC_ and 170.3 +/- 11.3 for HIV-1_NL4-3_ (n = 6; p<0.01, Mann-Whitney). Based on these parameters, the decrease in apparent infectivity of SIV_MAC_ did not appear to be explained by poor expression of the GFP reporter from the SIV LTR. The latter point was demonstrated more conclusively by using 3-part lentiviral vectors in which the GFP reporter was expressed from the HIV-1 and SIV_MAC_ vectors using an identical spleen focus-forming virus (SFFV) promoter (Fig 2B); the relative decrease in CRFK-normalized, SIV_MAC_ infectivity on Jurkat with the 3-part vector was at least as great in magnitude as it was with the 2-part vectors.

**Fig 3:**
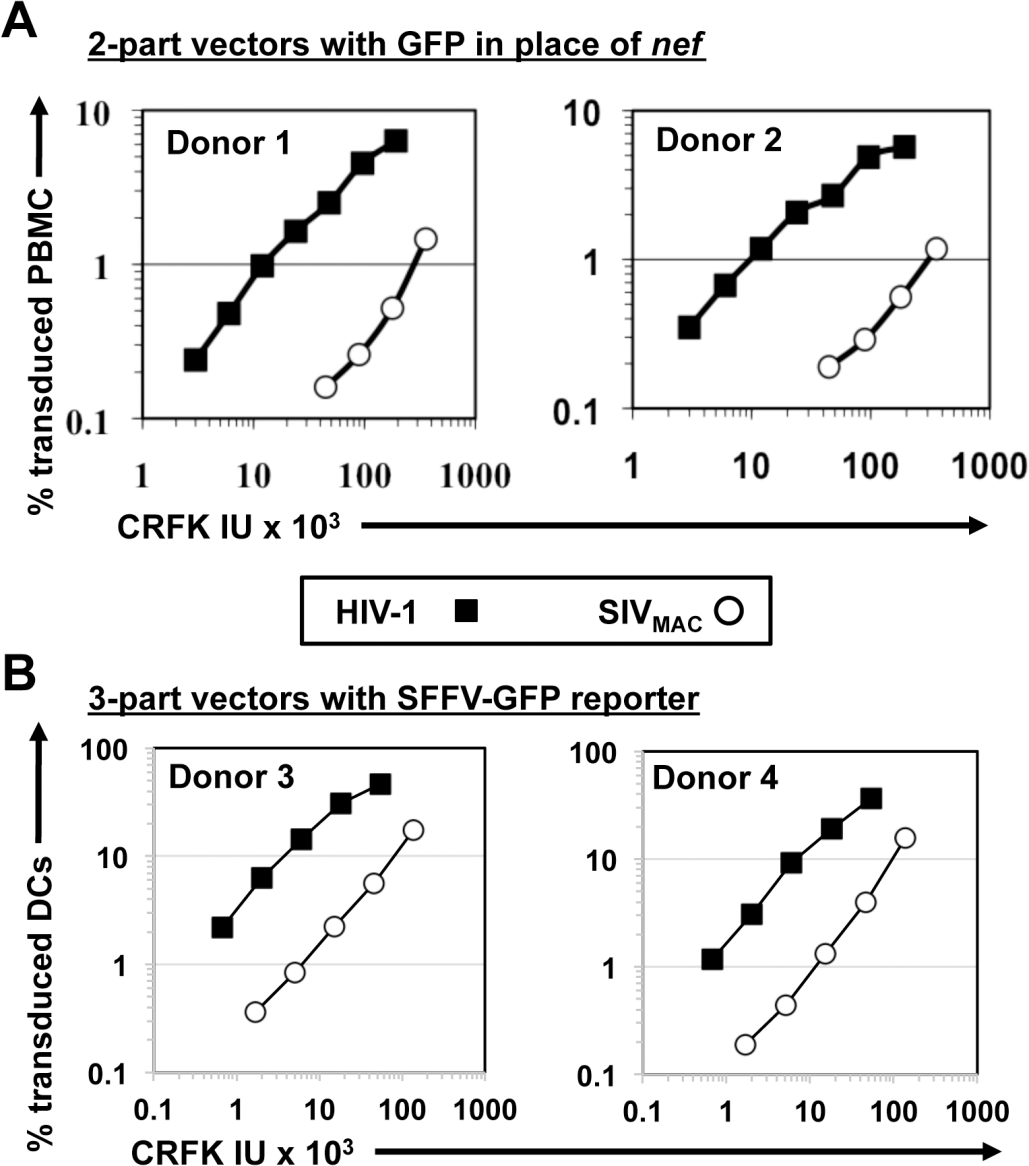
SIV_MAC_ transduction of human peripheral blood mononuclear cells or of monocyte derived dendritic cells is less efficient than by HIV-1. (A) VSV G-pseudotyped HIV-1_NL4-3_GFP (black squares) and SIV_MAC_239GFP (white circles) two-part vectors were generated by plasmid transfection of 293T cells. Vector stocks were normalized by titer on CRFK cells, and then used to challenge human peripheral blood mononuclear cells. (B) VSV G-pseudotyped, HIV-1_NL4-3_ (black squares) and SIV_MAC_239 (white circles) three-part vectors were generated by plasmid transfection of 293T cells. In each case, the viral genomic RNA was designed to transduce an identical SFFV-GFP reporter gene. Vector stocks were normalized by titer on CRFK cells, and then used to challenge monocyte derived dendritic cells (DCs). 2 days post-challenge, the percentage of GFP-expressing cells was determined by FACS. Data is plotted as percent GFP^+^ (infected) cells (Y axis) versus CRFK infectious units (IU) x 1,000 (X axis). Shown are representative data with cells from 4 independent blood donors.

Next, the infectivity of SIV_MAC_ was compared with that of HIV-1_NL4-3_ in primary human blood cells using the two-part vectors. Peripheral blood mononuclear cells (PBMCs) were prepared, stimulated with PHA for three days, and challenged with the single-cycle vectors. SIV_MAC_ transduction was less efficient than for HIV-1_NL4-3_ (Fig 3A). The magnitude of this difference was ~20-fold. Similar magnitude differences were observed when three-part vectors were used to challenge human, monocyte-derived dendritic cells in the presence of Vpx+-VLPs (Fig 3B). The dendritic cell experiments were carried out as previously described by providing SIV Vpx *in trans* using SIV VLPs [48–50].

**Fig 4:**
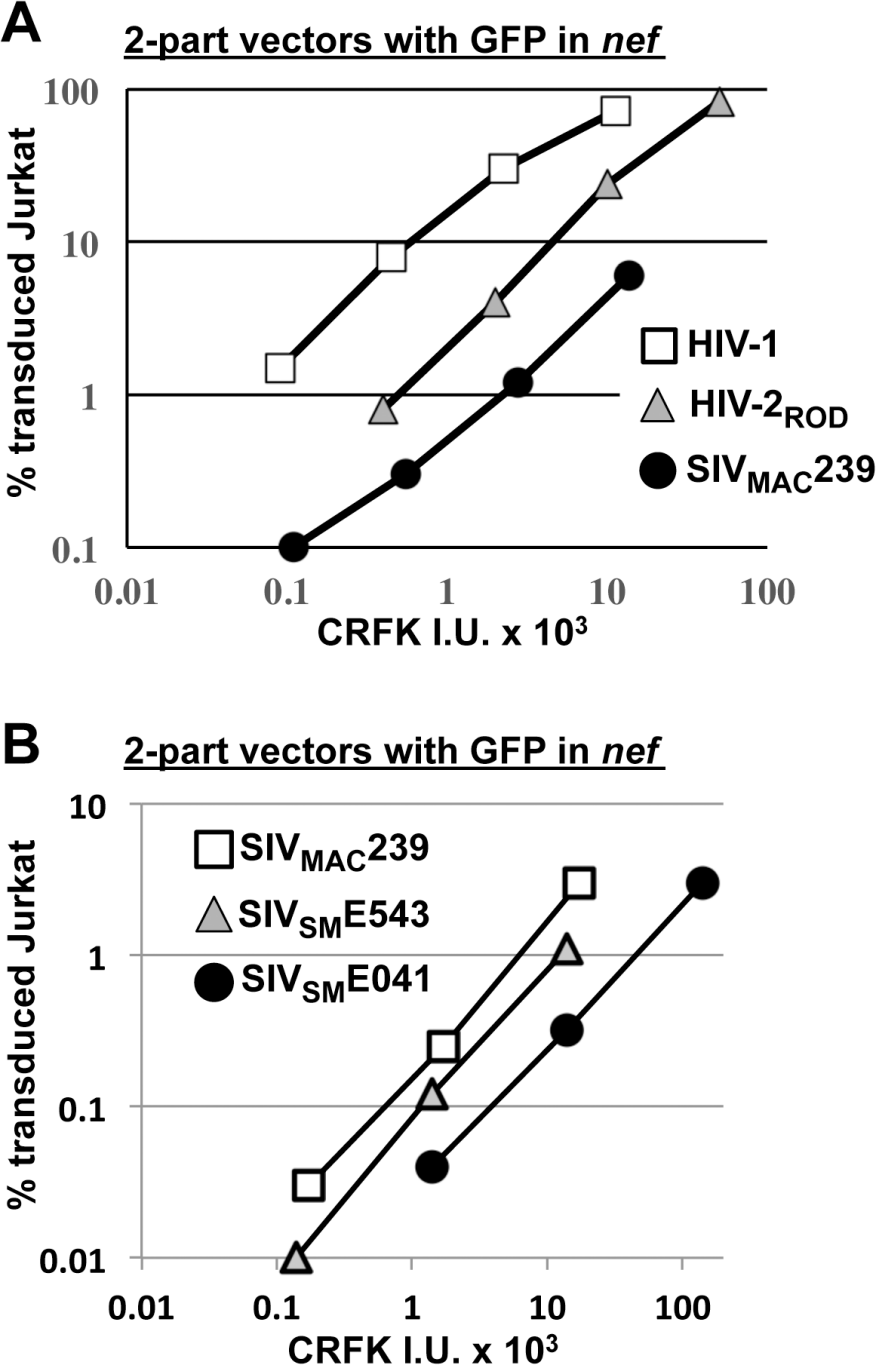
SIV_MAC_, HIV-2, and SIV_SM_ transduction of human T cells is less efficient than transduction by HIV-1. (A) Transduction efficiency of VSV G-pseudotyped two-part vectors for HIV-1_NL4-3_GFP (white squares), HIV-2_ROD_GFP (grey triangles), or SIV_MAC_239GFP (black circles) on Jurkat T cells. (B) Chimeric vectors were generated in which *gag-pol* of SIV_MAC_239GFP (white squares) was replaced with *gag-pol* from SIV_SM_E543 (grey triangles) or SIV_SM_041 (black circles). In each case (A and B), VSV G-pseudotyped vectors were generated by plasmid transfection of 293T cells. Vector stocks were normalized by titer on CRFK cells, and then used to challenge Jurkat T cells. 48 hrs post-challenge, the percentage of GFP-expressing cells was determined by FACS. Data is plotted as percent GFP^+^ (infected) cells (Y axis) versus CRFK infectious units (IU) x 1000 (X axis).

SIV_MAC_ and HIV-2 are believed to have arisen from cross-species transmission of SIV_SM_ from sooty mangabey monkeys to rhesus macaques and humans, respectively [1,2]. We therefore investigated to what extent other members of the SIV_SM_ lineage are capable of transducing Jurkat cells. An *env*-minus, VSV G-pseudotyped HIV-2_ROD_ vector, in which *nef* was replaced with GFP, was normalized to the HIV-1_NL4-3_GFP and SIV_MAC_239GFP vectors by transduction titer on CRFK and used to transduce Jurkat T cells. The normalized titer for SIV_MAC_ was roughly 20-fold lower than that for HIV-1_NL4-3_ on Jurkat cells (Fig 4A). HIV-2_ROD_ transduction was nearly 10-fold lower than HIV-1_NL4-3_ on Jurkat cells (Fig 4A).

SIV_MAC_239, the virus utilized in the experiments above, is highly adapted to rhesus macaques, having been passaged many times in these animals since the 1960s [13,51]. SIV_SM_E041 is a virus that was isolated directly from sooty mangabey monkeys [52]. SIV_SM_E543 was passed twice through rhesus macaques [53] and would therefore be expected to have a modest level of adaptation to the new host. As compared with SIV_MAC_239, a three-part vector generated from the non-adapted SIV_SM_E041 [54] had decreased Jurkat-specific transduction efficiency (Fig 4B). A three-part vector generated from SIV_SM_E543 [54], the SIV_SM_ virus that had been serially replicated in a non-native host (macaques), had transduction activity more similar to that of SIV_MAC_239 (Fig 4B). These results suggest that, in order to efficiently infect humans or rhesus macaques, SIV_SM_ must acquire resistance to a putative restriction activity present in blood cells.

### The capsid of SIV_MAC_, HIV-2, or SIV_SM_ is sufficient to decrease HIV-1 transduction efficiency in a T cell-specific manner

The experiments described above suggest that SIV_MAC_, SIV_SM_, and HIV-2 transduction is sensitive to a restriction activity that is elaborated by human blood cells. Since capsid (CA) is the retroviral determinant that confers sensitivity to several restriction factors, including Fv1 [17], TRIM5 [23,45], and Mx2 [55–57], the transduction efficiency of the 2-part HIV-1 vector described above was compared with that of an isogenic vector in which CA coding sequence was replaced with that from SIV_MAC_239 or HIV-2_ROD_. Neither of the two chimeras had transduction activity on CRFK cells or on HeLa cells.

**Fig 5:**
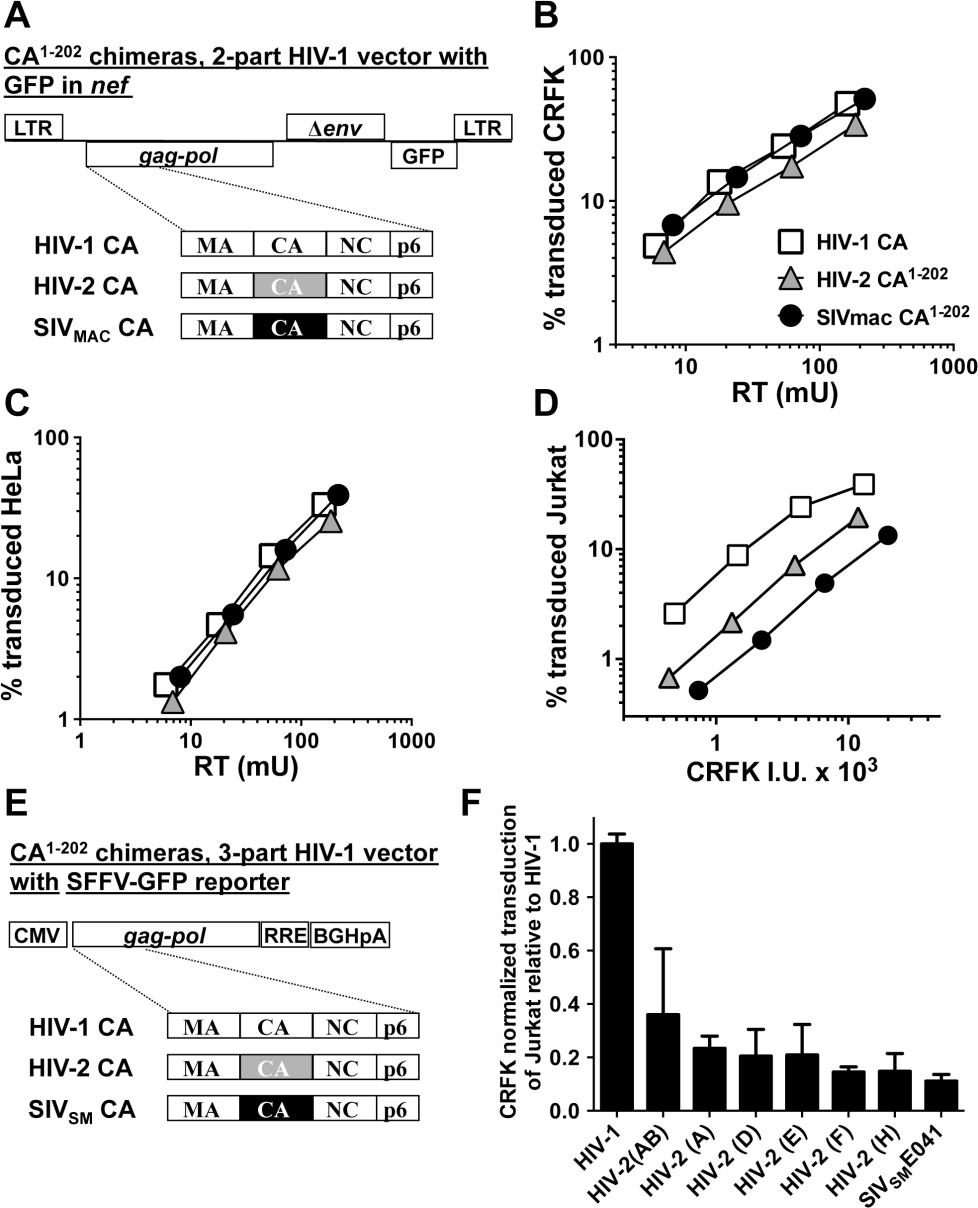
The capsid of SIV_MAC_, HIV-2, or SIV_SM_ is sufficient to decrease HIV-1 transduction efficiency in a T cell-specific manner. (A) Chimeric vectors were generated in which the coding sequence for HIV-1 CA amino acid residues 1 to 202 of the two-part HIV-1_NL4-3_GFP vector (white squares) was replaced with sequence encoding the corresponding amino acid residues from HIV-2_ROD_ (grey triangles) or SIV_MAC_239 (black circles). VSV G-pseudotyped vector was generated for each by transfection of 293T cells. Stocks were normalized by RT and used to challenge CRFK cells (B) or HeLa cells (C). Stocks were then normalized for CRFK transduction activity and used to challenge Jurkat T cells (D). 48 hrs post-challenge, the percentage of GFP-expressing cells was determined by FACS. Data is plotted as percent GFP^+^ (infected) cells (Y axis) versus RT activity (B and C), or versus CRFK infectious units (IU) x 1000 (X axis). (E) Chimeric vectors were generated in which the coding sequence for HIV-1 CA amino acid residues 1 to 202 of the HIV-1 *gag-pol* expression vector (white) was replaced with sequence encoding the corresponding amino acid residues from various HIV-2 isolates (grey) or SIV_SM_E041 (black). Three-part, VSV G-pseudotyped, SFFV-GFP bearing vectors were generated for each CA chimera by transfection of 293T cells. Stocks were then normalized for CRFK transduction activity and used to challenge Jurkat T cells. 48 hrs post-challenge, the percentage of GFP-expressing cells was determined by FACS. Data is plotted as CRFK normalized transduction of Jurkat cells, relative to the parental HIV-1 vector (F). Accession numbers for the different CA coding sequences are as follows: HIV-2(AB), 731744; HIV-2(A), GH123; HIV-2(D), L33083; HIV-2(E), L33087; HIV-2(F), U75441; HIV-2(H), AY5308; SIV_SM_E041, HM059825.

Since restriction factor sensitivity determinants are often located within the N-terminal two-thirds of CA [58], we then trimmed the C-terminal coding sequences of HIV-2_ROD_ and SIV_MAC_239 CA back to amino acid 202, using HIV-1 CA sequences to encode amino acids 203 to 230 (Fig 5A). When normalized by RT activity [46] the two chimeras exhibited transduction efficiency on CRFK and HeLa cells very similar to the parental vector (Fig 5B and 5C, respectively). In contrast, the chimeric vectors bearing SIV_MAC_239 or HIV-2_ROD_ CA transduced Jurkat T cells less efficiently, with a magnitude reduction that correlated with the respective parental vectors (Fig 5D).

Having established that CA from either SIV_MAC_239 or HIV-2_ROD_ is sufficient to reduce Jurkat T cell transduction efficiency by a 2-part HIV-1 vector (Fig 5D), fifteen additional chimeras were generated in the context of a 3-part HIV-1 vector using CA coding sequences from nine different HIV-2 Groups (Fig 5E). Many of the non-epidemic HIV-2 Groups in the database consist of single isolates, for which only partial HIV-2 CA sequences (encoding amino acids 1 to 162) are available [11]. In the case of these partial CAs, HIV-2_ROD_ sequence was substituted for the missing HIV-2 sequences (amino acids 163 to 202). As with the 2-part vectors, no infectivity was observed unless CA amino acids 203–230 were provided by HIV-1. Among the chimeras generated, representatives from Groups AB, A, D, E, F, and H, and from a primary SIV_SM_, were sufficiently infectious to evaluate CRFK-normalized transduction efficiency on Jurkat T cells. As a general trend, chimeras generated with CA from the epidemic Groups (A and B) were the most infectious on Jurkat T cells, those from the non-epidemic Groups (D, E, F, and H) were less infectious, and that from SIVSM was the least infectious (Fig 5F). These results suggest that SIV_SM_ must acquire resistance to the putative CA-specific restriction activity present in human blood lymphoid cells in order to efficiently infect human blood cells.

### The defect in Jurkat transduction associated with SIV_MAC_ CA is independent of the viral entry pathway

**Fig 6:**
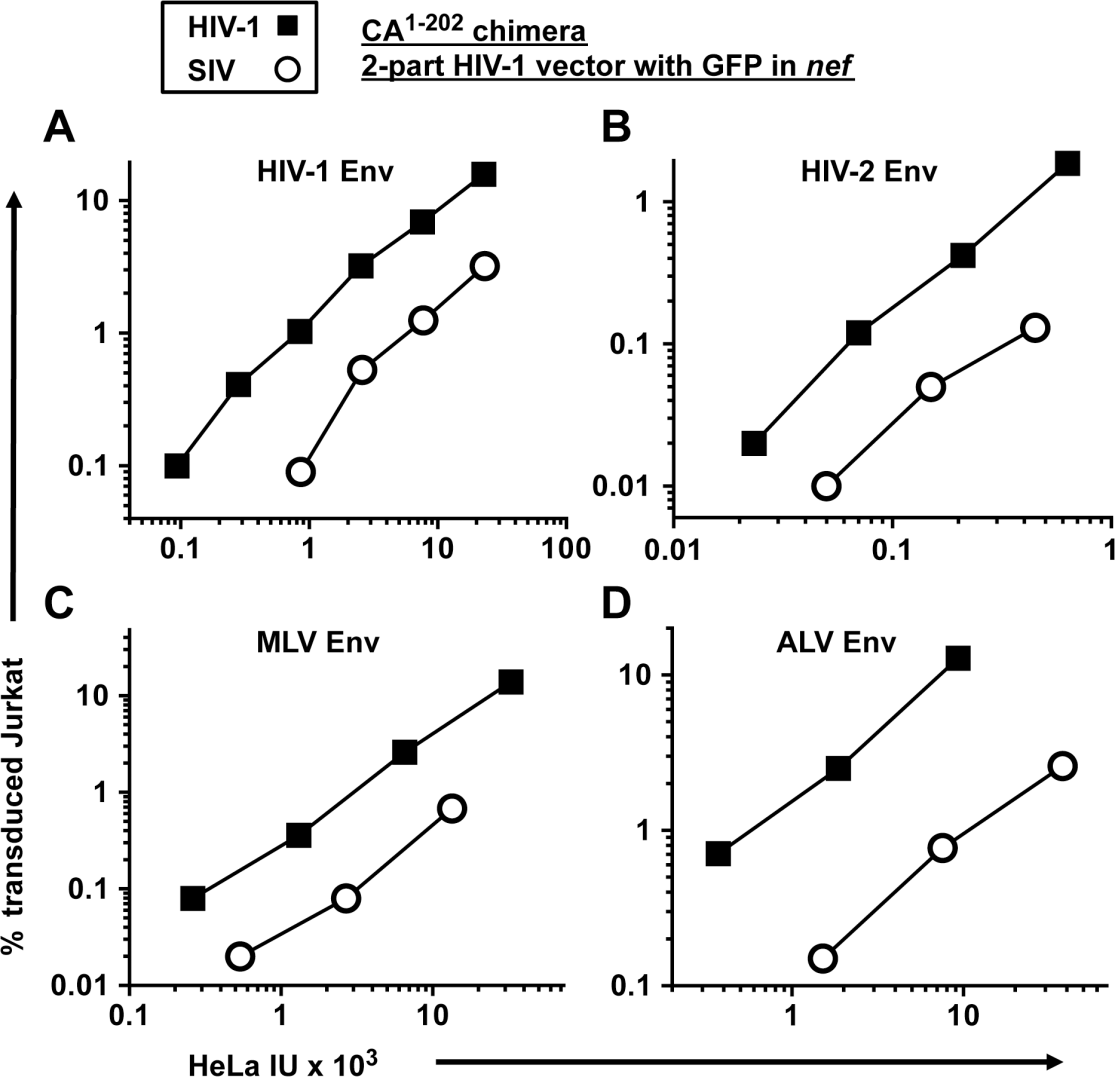
The transduction defect associated with SIV_MAC_ CA is independent of the virus entry pathway. A two-part, *env*-minus HIV-1 vector with GFP in place of nef (black squares), or an isogenic vector in which CA^1-202^ coding sequences were replaced with those from SIV_MAC_239 (white circles), were produced by 293T transfection. Each vector was pseudotyped with Env glycoprotein from either HIV-1_HXB2_(A), HIV-2_MCN_ (B), ecotropic MLV (C), or ALV-A (D) and transduction efficiency was measured on HeLa cells bearing human CD4 (A and B), the mCAT1 ecotropic receptor (C), or the avian TVA receptor (D), and then used to challenge Jurkat cells bearing the same receptors. 48 hrs post-challenge, the percentage of GFP-expressing cells was determined by FACS.

All of the experiments above were conducted with vectors pseudotyped with VSV G. To determine if the decreased transduction efficiency associated with the SIV_MAC_ CA is observed with other glycoproteins, a two-part, *env*-minus HIV-1 vector with GFP in place of *nef*, or an isogenic vector in which CA^1-202^ coding sequences were replaced with those from SIV_MAC_239, were pseudotyped with Env glycoproteins from either HIV-1_HXB2_, HIV-2_MCN_, ecotropic MLV, or ALV-A (Fig 6). The transduction titer of each pseudotyped vector was first measured on HeLa cells bearing either human CD4, murine mCAT1 ecotropic receptor, or avian TVA receptor. Each was then used to challenge Jurkat T cells that had been stably transduced to bear the cognate receptors, as appropriate. 48 hrs post-challenge, the percentage of GFP-expressing cells was determined by FACS. In each case, the chimeric vector bearing SIV_MAC_ CA^1-202^ was as defective as the VSV G-pseudotyped vector (Fig 6A–6D). These results demonstrate that the Jurkat transduction defect associated with the SIV_MAC_ CA is independent of the pathway of viral entry.

### As_2_O_3_ increases SIV_MAC_ transduction of human blood cells

Given the results described above, evidence was sought that the cell type-specific defect in SIV_MAC_ transduction efficiency might be due to a dominant-acting, human blood-specific, restriction factor. Restriction activity of the capsid-specific restriction factors Fv1 and TRIM5 is saturated by large quantities of virus-like particles (VLPs) bearing restriction-sensitive cores [17]. Flat, epithelial cells work well as viral targets in TRIM5 saturation experiments; in contrast, saturation experiments have not been possible in T cell lines [59,60]. Attempts here to saturate putative SIV_MAC_-specific restriction activity in Jurkat T cells with SIV VLPs were also unsuccessful.

As_2_O_3_ rescues retroviruses from CA-specific restriction by TRIM5 but has no effect on retrovirus transduction efficiency in the absence of TRIM5-mediated restriction [31,35,47,61,62]. The exact mechanism by which As_2_O_3_ blocks TRIM5-mediated restriction is not known, though the effect results in increased reverse transcription and correlates with disruption of mitochondrial membrane potential [31,61].

**Fig 7:**
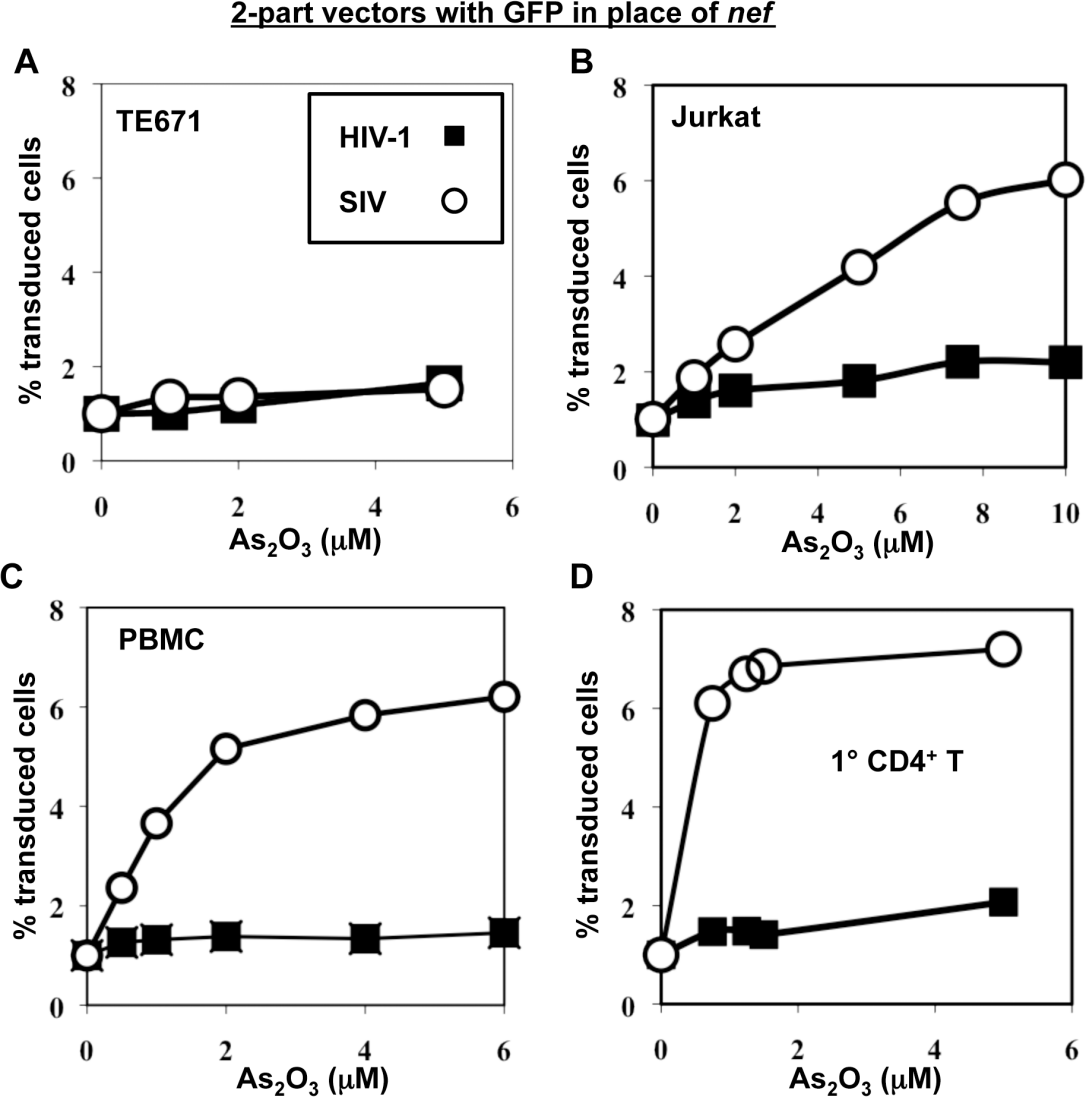
As_2_O_3_ specifically increases SIV_MAC_ infectivity in human blood cells. TE671 cells (A), Jurkat T cells (B), human PBMC (C), or human CD4^+^ T cells (D) were transduced with two-part, VSV G-pseudotyped HIV-1_**NL4-3**_-GFP or SIV_**MAC**_GFP vectors using a predetermined quantity of virus such that 1% of cells were infected. As_**2**_O_**3**_ was added 1 hr prior to vector challenge and maintained for 12 hrs post-infection, at the concentrations indicated on the X axis. 48 hrs post-challenge the percentage of GFP-expressing cells was determined. The Y axis shows the fold increase relative to infection without As_**2**_O_**3**_.

To test the hypothesis that SIV_MAC_ transduction of human blood cells might be restricted by TRIM5, or by a cellular factor with similar properties, the effect of As_2_O_3_ on SIV_MAC_ transduction was assessed. As_2_O_3_ had no effect on the transduction efficiency of VSV G-pseudotyped, 2-part vectors for either SIV_MAC_239 or HIV-1_NL4-3_ in TE671 (Fig 7A), an adherent rhabdomyosarcoma cell line in which SIV_MAC_ infectivity was equivalent to that of HIV-1_NL4-3_ (Fig 1). In contrast, As_2_O_3_ increased SIV_MAC_ transduction of Jurkat T cells 3-fold, and transduction of PBMCs or primary CD4^+^ T cells 7-fold (Fig 7B–7D). HIV-1_NL4-3_ T cell transduction of any of these cells was increased less than 2-fold by As_2_O_3_ (Fig 7B–7D). Thus, As_2_O_3_ enhanced SIV_MAC_ transduction of human blood cells in which relative transduction efficiency of SIV_MAC_ was compromised. These results are consistent with the presence of a TRIM5-like, SIV_MAC_-specific, restriction factor in human blood cells.

### SIV_MAC_ transduction efficiency in human CD4^+^ T cells does not increase with disruption of endogenous TRIM5α or CypA

**Fig 8:**
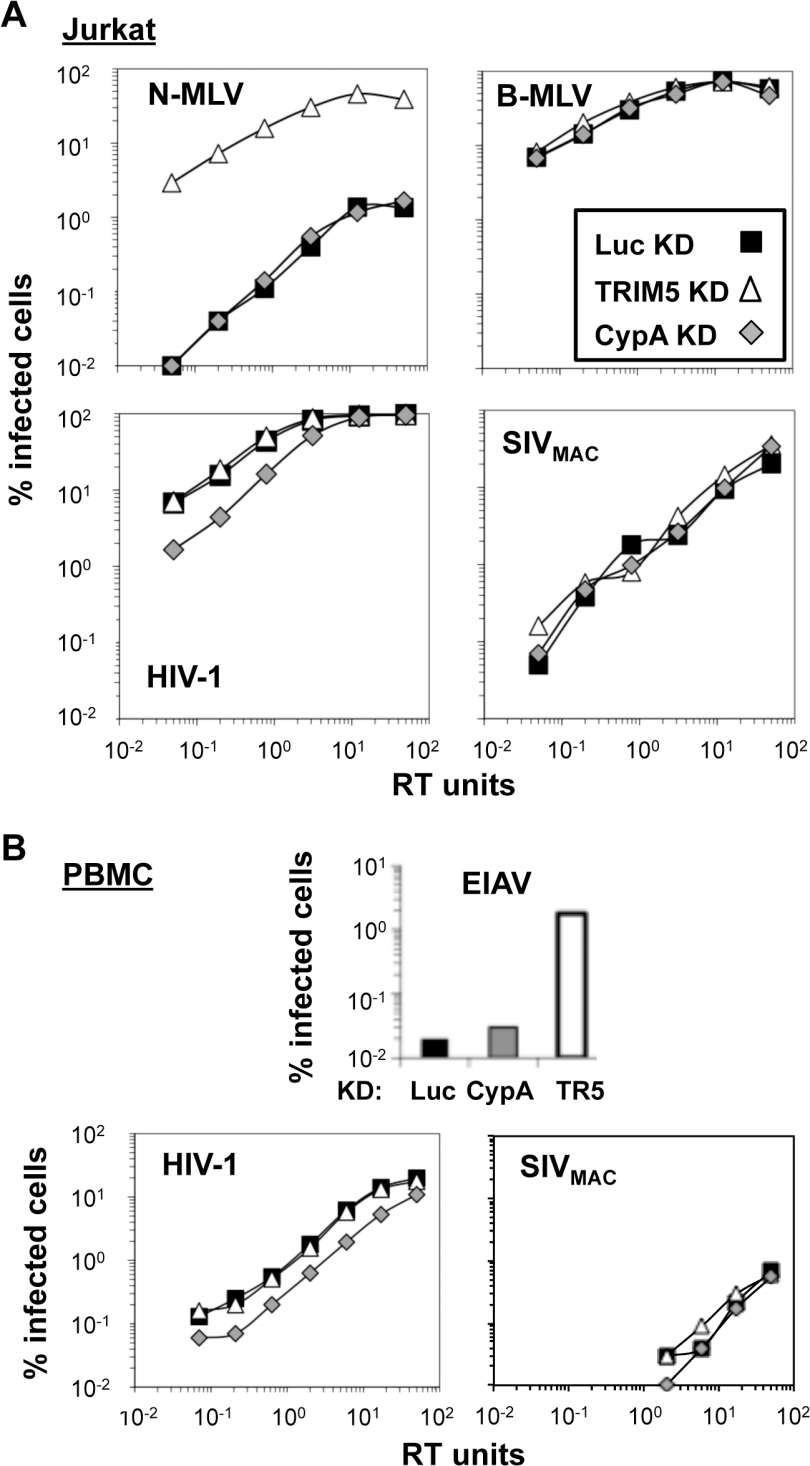
Knockdown of TRIM5 or of cyclophilin A has no effect on SIV_MAC_ transduction of Jurkat CD4^+^ T cells. Jurkat T cells (A) or primary human CD4^+^ T cells (B) were transduced with lentiviral vectors bearing a puromycin resistance cassette and miR30-based knockdown cassettes targeting either luciferase (black squares), CypA (gray diamonds), or TRIM5 (white triangles). Puromycin-resistant pools of transduced cells were challenged with VSV G-pseudotyped N-MLV_GFP_, B-MLV_GFP_, HIV-1_NL-GFP_, SIV_mac-GFP_, or EIAV_GFP_, as indicated. The percentage of GFP^+^ (infected) cells at 48 hrs is reported. HIV-1_NL-GFP_ and SIV_mac-GFP_ vectors were two-part vectors, with GFP in place of *nef*. N-MLV_GFP_, B-MLV_GFP_, and EIAV_GFP_ were three-part vectors.

TRIM5 is a well-characterized host cell restriction factor that decreases retroviral transduction in a capsid-specific fashion [23,45]. Though ectopic expression of human TRIM5α in adherent cell lines shows minimal restriction activity against SIV_MAC_ [23,34] it was important to determine whether endogenous human TRIM5α contributes to the SIV_MAC_ transduction block in human blood cells. To investigate this possibility, a miR30-based TRIM5 knockdown cassette was delivered to Jurkat T cells using a lentiviral vector as previously described [48,63,64] (Fig 8). The vector also expresses a puromycin-resistance gene that was exploited to select pools of transduced cells. Cyclophilin A (CypA), an HIV-1 capsid binding protein [65] that promotes TRIM5-mediated restriction in some cell types [66], and appears to protect against an unknown restriction activity in other cells [17], was also targeted for knockdown with a lentiviral vector. As a control for miR30 lentiviral vector transduction and puromycin selection, Jurkat T cells were transduced with an otherwise isogenic lentiviral vector targeting luciferase (Luc), a gene that is not present in these cells.

TRIM5 knockdown efficiency in Jurkat T cells cannot be assessed by western blot since endogenous human TRIM5 is not detectable in these cells using available antibodies. Instead, knockdown efficiency can be deduced by comparing the infectivity of a pair of viruses, one of which is restricted by human TRIM5 (N-MLV), and the other which is not restricted (B-MLV) [59]. The three pools of puromycin-resistant Jurkat T cells–either knocked down for TRIM5, CypA, or Luc—were therefore challenged with N-tropic or B-tropic MLV-GFP reporter viruses. As shown previously [59], N-tropic MLV was much less infectious than B-tropic MLV in the control (luciferase) knockdown cells (Fig 8A). TRIM5 knockdown increased N-MLV transduction efficiency up to the level achieved by the non-restricted B-tropic MLV (Fig 8A) but no effect on the transduction efficiency of HIV-1_NL4-3_ or SIV_MAC_ was observed (Fig 8A). Also, as shown previously (3, 4), knockdown of CypA had no effect on N-tropic MLV, B-tropic MLV, or SIV_MAC_ (Fig 8A), though CypA knockdown decreased HIV-1_NL4-3_ transduction efficiency by 3 to 4-fold (Fig 8A). Thus, the low relative transduction of Jurkat T cells by SIV_MAC_ was not increased by knockdown of TRIM5 or CypA.

To extend these findings to primary cells, human CD4^+^ T cells were enriched from peripheral blood by positive-selection with magnetic beads, stimulated with plate-bound anti-CD3 and anti-CD28 antibodies, and transduced with the same lentiviral vectors for stable knockdown of TRIM5, CypA, or luciferase, as previously described [67] (Fig 8B). Transduced cells were propagated in puromycin-resistant pools. Transduction with a control vector in which the puromycin resistance cassette was replaced with GFP demonstrated that primary transduction efficiency, in the absence of drug selection, was greater than 90%. Growth of transduced CD4^+^ T cells in tissue culture was maintained in an ongoing fashion by TCR re-stimulation every two weeks [67].

CD4^+^ T cells from one of two representative blood donors are shown in Fig 8B. The titer of the N-tropic and B-tropic MLV vectors on the stably-transduced, primary human CD4^+^ T cells, was not sufficient to assess the efficiency of TRIM5 knockdown. Instead, a lentiviral vector derived from the equine infectious anemia virus (EIAV-GFP) was utilized [68]. As previously shown in human HeLa cells [34], knockdown of TRIM5 increased EIAV-GFP transduction efficiency (Fig 8B). Though the absolute infectivity of EIAV-GFP in the luciferase and CypA knockdown cells was at the limit of detection, it was possible to document an increase in EIAV-GFP transduction efficiency of at least 50-fold in the TRIM5 knockdown cells, confirming that the TRIM5 knockdown was robust. As expected, CypA knockdown caused a modest reduction in HIV-1_NL4-3_ infectivity (Fig 8B). SIV_MAC_ was 50- to 100-times less infectious than HIV-1_NL4-3_ in all of the CD4^+^ T cell knockdown lines tested (Fig 8B). Thus, neither TRIM5 knockdown nor CypA knockdown increased SIV_MAC_ transduction efficiency, in Jurkat T cells or in primary CD4+ T cells.

### The block to SIV_MAC_ transduction in Jurkat T cells occurs prior to establishment of the provirus, but after entry into the target cell nucleus

**Fig 9:**
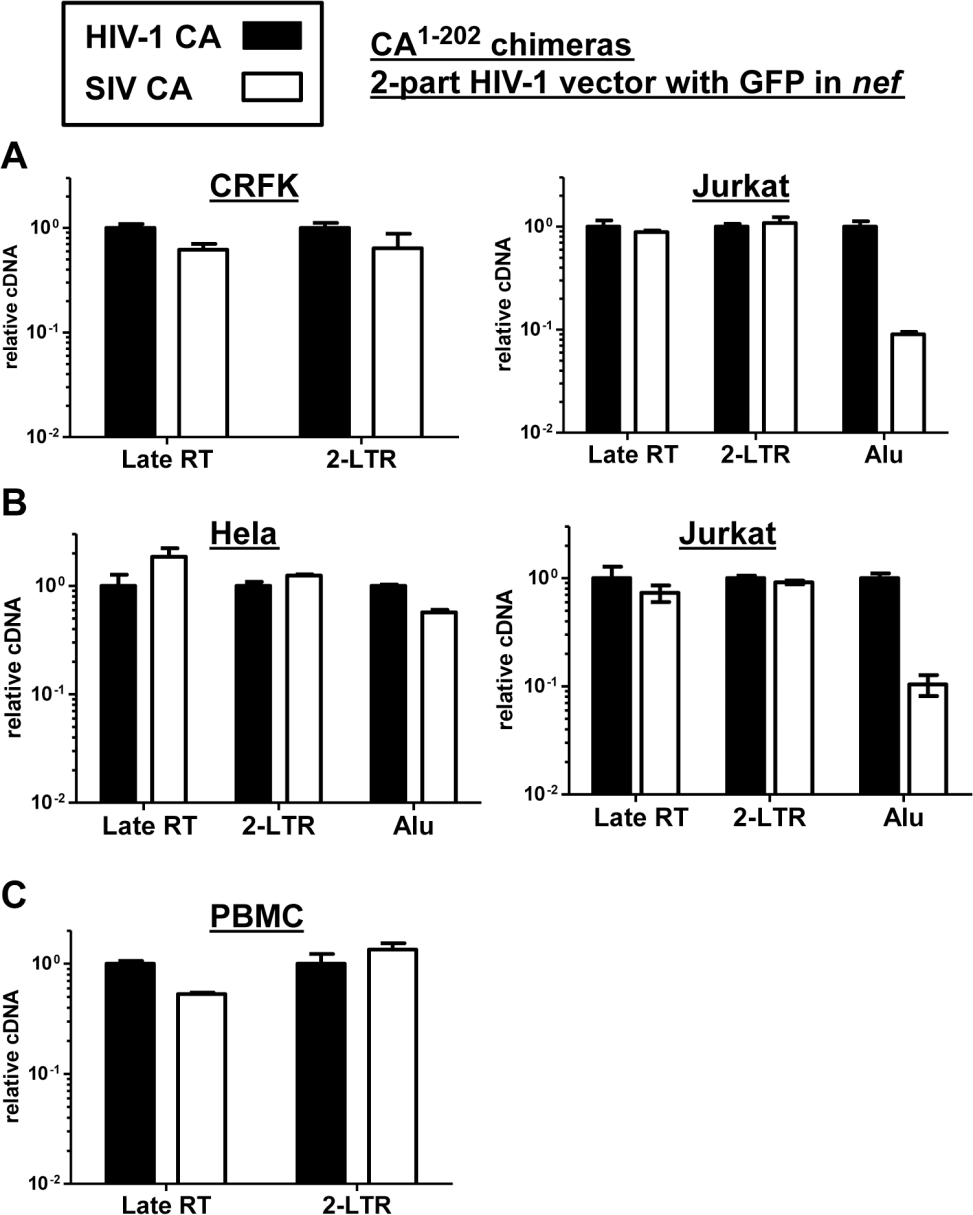
The block to SIV_MAC_ infection of Jurkat T cells occurs after formation of 2-LTR circles. CRFK and Jurkat (A), or Hela and Jurkat (B), or PBMCs (C) were infected with VSV G-pseudotyped HIV-1_NL4-3_-GFP, or with isogenic vector bearing the SIV_MAC_239 CA residues 1 to 202. 24 hrs post-infection, DNA was collected from the cells and subjected to qPCR using primers specific for full-length linear viral cDNA, 2-LTR circles, or proviral DNA, as indicated. Shown is the abundance of signal from vector bearing the SIV_MAC_239 CA^1-202^, relative to the amount of signal from HIV-1_NL4-3_-GFP. In each case, infection was performed in the presence of an RT inhibitor to control for background levels of signal.

To determine where in the retroviral replication cycle the relative block to SIV_MAC_ transduction occurs, CRFK cells and Jurkat T cells were challenged with the single-cycle, 2-part, HIV-1_NL4-3_ GFP reporter vector, or the isogenic vector bearing the SIV_MAC_239 CA, that were diagramed schematically in Fig 5A. Full-length linear viral cDNA, 2-LTR circle viral cDNA, and proviral DNA as assessed by Alu-PCR were quantitated by real-time PCR, using previously described protocols [69,70]. The relative level of PCR product obtained with the vector bearing SIV_MAC_239 CA was expressed as a percentage of that obtained with the vector bearing HIV-1_NL4-3_ CA, with the latter set at 100%. In CRFK cells, infection with the two vectors resulted in comparable amounts of full-length linear and 2-LTR circles (Fig 9A). As compared with the vector bearing HIV-1_NL4-3_ CA, transduction of Jurkat T cells with the vector bearing SIV_MAC_239 CA resulted in the same amount of full-length linear cDNA and 2-LTR circles, but 10-fold less product for Alu-PCR (Fig 9A).

Since Alu repeats are primate-specific [71], Alu-PCR could not be performed using the feline CRFK cells as transduction targets. Therefore, similar experiments were performed with HeLa cells (Fig 9B). In addition, a PCR protocol for 2-LTR circles was used in which one of the PCR primers spans the circle junction; this distinguishes bona fide 2-LTR circles from auto-integrants [72]. No defect in full-length linear cDNA or 2-LTR circles was detected when transduction of Jurkat cells with the vector bearing SIV_MAC_239 CA was compared with the vector bearing HIV-1_NL4-3_ CA (Fig 9B). As compared with HeLa cells, a specific defect in provirus establishment in Jurkat T cells by the vector bearing SIV_MAC_239 CA was observed (Fig 9B). Similar results were obtained using human PBMCs as target cells, though the signal from Alu-PCR was insufficient to quantitate the magnitude difference between HIV-1 and SIV_MAC_ (Fig 9C). These results indicate that reverse transcription and nuclear transport by particles bearing SIV_MAC_ CA is equivalent to that of particles bearing HIV-1_NL4-3_ CA, and that the relative block to SIV_MAC_ transduction likely occurs after entry into the nucleus, prior to integration.

### Poor relative infectivity of SIV_MAC_239 in human blood cells results from a dominant-acting restriction activity

Human blood cells such as Jurkat T cells might be less permissive for SIV_MAC_ transduction because they lack a factor, which is present in epithelial cell lines such as HeLa, that promotes SIV_MAC_ transduction. Alternatively, human blood cells might possess an inhibitor of SIV_MAC_ transduction that is absent from the adherent cell lines. To determine which of these two possibilities is correct, Jurkat T cells were fused with HeLa cells using polyethylene glycol. The resulting heterokaryons were then challenged with the single-cycle, HIV-1_NL4-3_ GFP reporter vector (hCA-GFP), or the isogenic vector bearing the SIV_MAC_239 CA (sCA-GFP), that were shown schematically in Fig 5A.

**Fig 10:**
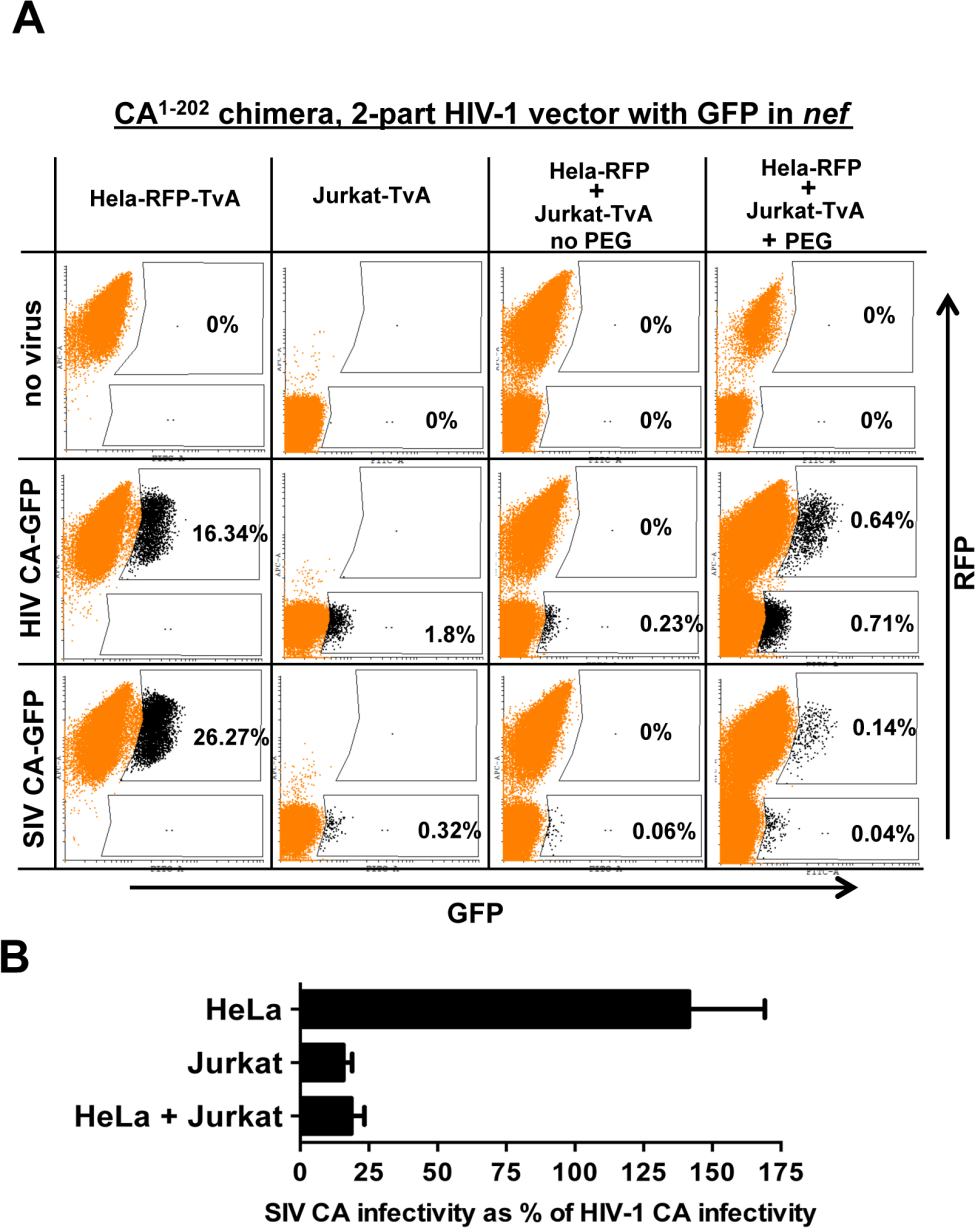
Evidence for a dominant-acting, capsid-specific, restriction activity in Jurkat T cells. (A) Jurkat and HeLa cells stably expressing the ALV-A receptor (TvA) or TagRFP-657, as indicated, were fused by treatment with PEG and transduced with ALV-A Env-pseudotyped HIV-1_NL4-3_-GFP, or with isogenic vector bearing the SIV_MAC_239 CA^1-202^. Shown are flow cytometry dot plots obtained 48 hrs post-transduction. HeLa-TagRFP-657 cells are only permissive to infection with ALV-A Env-pseudotyped vectors after fusion with Jurkat-TvA. Infected heterokaryons were visualized as GFP and TagRFP-657 double-positive cells. As a positive transduction control, TagRFP-657 and TvA were also co-expressed in HeLa cells, as indicated. The percentage of transduced cells are indicated. (B) Bar graph showing the infectivity of the SIV_MAC_239 CA^1-202^-bearing vector relative to the isogenic vector bearing HIV-1 CA, for the HeLa, Jurkat and heterokaryons. Data from the flow cytometry data shown in A, and two repeat experiments, is shown with the standard deviation.

A flow cytometry-based assay was established that discriminates infected heterokaryons from those cells that fail to form heterokaryons (Fig 10). Primary flow cytometry data for a single representative experiment is shown in Fig 10A; Fig 10B shows a bar plot of the results for three independent experiments. The HeLa cells that were used in the fusion stably synthesized TagRFP-657, a far-red fluorescent protein [73]. The Jurkat T cells that were used in the fusion stably bore the avian leukosis virus TvA receptor on their surface. The HIV-1 CA-GFP and SIV CA-GFP vectors were pseudotyped with avian leukosis virus subtype A (ALV-A) Env so that the vectors were able to enter Jurkat-TvA cells but not the HeLa-RFP cells. Heterokaryons formed by fusion of the two cell types would bear the cognate receptor for ALV-A Env and would also be positive for RFP. Infected heterokaryons, then, would be positive for GFP and RFP.

As a control, HeLa-RFP cells were engineered to express TvA (HeLa-RFP-TvA); in these cells, transduction with SIV CA-GFP was 1.6-fold higher than with HIV-1 CA-GFP (Fig 10A). Challenge of the Jurkat-TvA cells with HIV-1 CA-GFP and SIV CA-GFP recapitulated the phenotype of the parental Jurkat cells. That is, transduction of Jurkat-TvA cells with SIV CA-GFP was 5.6-fold less efficient than with HIV-1 CA-GFP, though, when these values are corrected for the transduction efficiency on HeLa-TVA, the difference is 9-fold (Fig 10A). When Jurkat-TvA cells were mixed with HeLa-RFP cells in the absence of polyethylene glycol, no GFP/RFP double-positive cells were detected, and transduction with SIV CA-GFP was 6-fold (corrected) less efficient than with HIV-1 CA-GFP (Fig 10A). When Jurkat-TvA cells were mixed with HeLa-RFP cells in the presence of polyethylene glycol, GFP/RFP double positive cells were detected, and transduction of this population with SIV CA-GFP was 7.3-fold (corrected) less efficient than with HIV-1 CA-GFP (Fig 10A). The bar graph in Fig 10B shows the results for three experiments with the standard deviation. The results of this heterokaryon assay indicate that Jurkat T cells possess a dominant-acting restriction activity specific for SIV CA.

## Discussion

The characteristics of a previously unreported retroviral restriction activity in human blood cells are described here. The first clue to the existence of this restriction activity was that SIV_MAC_239 transduced human blood cells less efficiently than did HIV-1. Lower SIV_MAC_239 transduction efficiency relative to HIV-1 was observed with all human blood-derived cells tested here, including cell lines of lymphoid and myeloid lineage, human PBMCs and primary CD4^+^ T cells, and, as previously described [49,50,74], monocyte-derived dendritic cells and macrophages. In contrast to blood-derived cells, fibroblasts, fibrosarcoma, epithelial carcinoma, and glioblastoma cell lines were transduced as efficiently by SIV_MAC_239 as by HIV-1.

The presence of a dominant-acting, SIV_MAC_239-specific, restriction factor in human blood cells—as opposed to the lack of a cofactor for SIV_MAC_239 replication in these cells—was supported by the finding of a block to SIV_MAC_239 replication in Jurkat/HeLa-heterokaryons of equal magnitude to the block in Jurkat T cells. Similar heterokaryon experiments demonstrated the presence of a dominant restriction activity prior to the cloning of the retroviral restriction factors APOBEC3G, TRIM5, and TETHERIN [42,43,75,76]. Several methods for quantitating transduction of heterokaryon target cells were tried here, all of which gave similar qualitative results. Of these assays, the heterokaryon assay presented in Fig 10 gave us the clearest assessment of heterokaryon transduction efficiency; it exploits the specificity of the ALV/TVA interaction [77], and the clean spectral separation of GFP from the far-red fluorescent protein TagRFP-657 [73].

As is the custom for naming dominant-acting, lentiviral restriction activities of unknown identity [40,43,78,79], the SIV_MAC_239-specific restriction activity described here will be called Lv4. Whether this activity is due to a single factor, or due to a multi-factor complex, remains to be determined. Knockdown experiments presented here showed that Lv4 is distinct from TRIM5 (Fig 8), the protein responsible for Lv1 activity [23,45]. Lv2 is an HIV-2 *env*-specific restriction activity [80]. Lv4 restricts vectors that are pseudotyped with VSV G or with Env from Lv2-resistant HIV-2 clone MCN (Fig 6) so it must be distinct from Lv2. For that matter it also restricts vectors pseudotyped with HIV-1 Env, MLV ecotropic Env, or ALV-A Env (Fig 6) so it acts independent of the viral entry pathway. Lv3 restricts HIV-1 in an *env*-specific fashion [79] and so it must also be distinct from Lv4.

SIV_MAC_239 CA was sufficient to transfer Lv4-sensitivity when it was substituted for HIV-1 CA (Fig 5). This observation puts Lv4 in good company with a growing family of restriction factors that target the retroviral CA. The CA-specific restriction factors Fv1 and TRIM5 can be saturated by virus-like particles (VLPs) bearing restriction-sensitive CA [40,43,81,82]. Attempts to saturate Lv4 with SIV_MAC_239 virus-like particles were unsuccessful, though this result was not unexpected since Lv4 was only observed in blood cells, and saturation of CA-specific restriction activities in non-adherent cells that grow in suspension has not been reported [59].

Others have shown that SIV transduces human T cell lines less efficiently than HIV-1 and they provided suggestive evidence that this difference was independent of TRIM5 and CypA [83]. Here, after demonstrating that Lv4 activity does not require TRIM5 through knockdown experiments in either Jurkat or PBMC (Fig 8), our attention was directed to other potential CA-specific candidates. Disruption of TNPO3 results in accumulation of the CA-binding protein CPSF6 in the cytoplasm and an associated block to HIV-1 nuclear entry [72]. Though inhibition of SIV_MAC_ by CPSF6 was slightly greater than that of HIV-1 [84], this differential effect was much smaller than was observed with Lv4. Additionally, Lv4 blocks SIV_MAC_ at a later stage in the lentiviral life cycle than does CPSF6 (Fig 9), as demonstrated using the same assay for *bona fide* HIV-1 2-LTR circles [72]. In response to the identification of MX2 in a targeted screen for HIV-1 inhibitors among interferon stimulated genes (ISGs) [85], and prior to identification of MxB as a lentivirus CA-specific inhibitor [55–57], MX2 was found to inhibit HIV-1 and SIV equally well when ectopically expressed in either HT1080 or HeLa cells, and thus ruled out as Lv4.

Like the restriction activity conferred by TRIM5 [47,61,67], Lv4 was suppressed by arsenic (Fig 5). Efficient knockdown of TRIM5 in Jurkat T cells or in primary CD4^+^ T cells, though, had no effect on SIV_MAC_ titer (Fig 8), indicating that Lv4 is distinct from TRIM5. How arsenic works to suppress restriction activity is not known. Among its many effects, arsenic inhibits NFκB signaling by oxidizing a critical cysteine in IKKα/β [86]. This suggests that arsenic might inhibit TRIM5 restriction activity by oxidizing critical cysteines. The fact that Lv4 is inhibited by arsenic suggests that, like TRIM5, it too might be a cysteine-containing protein. Attempts to identify the host factor responsible for Lv4 activity by ectopically expressing a panel of 36 TRIM family members [87], each of which possess cysteine-rich zinc-fingers and b boxes, has so far failed to identify an SIV_MAC_-specific inhibitor. That being said, the cell type-specific suppression of TRIM5 restriction activity by arsenic [47] suggests that arsenic targets a common cellular co-factor required for TRIM5 and Lv4 restriction activity. Such a co-factor might be an innate immune signaling molecule like those shown to be required for TRIM5-mediated restriction [48].

TRIM5 blocks retroviruses soon after entry into the cell cytoplasm [88]. This is evident as a block to the accumulation of viral cDNA [23]. If this block to reverse transcription is removed by arsenic or by proteasome inhibitors, a downstream block is encountered at the level of nuclear transport, with a decrease in viral cDNA circles [30,31]. The capsid binding proteins MX2 and, conditionally, CPSF6, both appear to block infection prior to entry in the nucleus [56,57,72,89]. The block due to Lv4 occurred before integration, but after completion of reverse transcription and nuclear entry, as indicated by levels of nascent viral cDNA, viral cDNA circles, and Alu-PCR (Fig 9). Thus, any putative factor underlying Lv4 activity likely interacts with CA within the nucleus and acts to block integration. These results are consistent with the steadily increasing evidence, acquired over many years, that CA plays an essential role within the nucleus of newly infected cells [90–93].

Finally, sensitivity to Lv4 was not unique to SIV_MAC_ but shared by other viruses in the same family, including HIV-2 and SIV_SM_ (Fig 4). Most studies here were performed with SIV_MAC_ because the restriction activity was more robust than for HIV-2, but it was not so severe as for SIV_SM_, which precluded quantitation of restriction activity against the latter virus. The relative restriction activity targeting these viruses is consistent with a model in which replication of HIV-2 necessitated adaption of the SIV_SM_ CA, such that it became relatively resistant to Lv4. There was indeed a trend such that HIV-2 isolates from non-epidemic Groups were generally more sensitive to Lv4 than were epidemic HIV-2 strains (Fig 5F). Though HIV-2 infects humans, relative to HIV-1 this virus is still restricted by Lv4. Thus, Lv4 may contribute to the fact that HIV-2-infected individuals are less likely to progress to AIDS than are those people infected with HIV-1 [94].

## Materials and Methods

### Plasmid DNAs

HIV-1_NL4-3_GFP, SIV_MAC_239GFP, HIV-2_ROD_GFP, SIV_SM_E041GFP, and SIV_SM_E543GFP encode modified proviral clones for the respective viruses [31,45,54,95]; each of these plasmids lacks functional *env* and encodes GFP instead of Nef. For some experiments, coding sequences for residues 1 to 202 of HIV-1_NL4-3_GFP were replaced by overlapping PCR with the corresponding CA coding sequences from HIV-2_ROD_, SIV_MAC_239, SIV_SM_E041 or SIV_SM_E543 [54,96,97]. CA^1-202^ chimeras were also generated within the context of p8.9NdSB [31,45,54,95]; the restriction sites BlpI and BstEII were introduced flanking CA coding sequences and the following sequences, synthesized by GenScript, were inserted at these restrictions sites:

>HIV-2(AB), 731744

GCTCAGCAAGCAGCAGCTGACACAGGAAACAACAGCCAGGTCAGCCAAAATTACCCAGTGCAACAAGTAGCTGGCAATTATGTCCATGTGCCGTTAAGTCCCCGAACCTTAAATGCCTGGGTAAAATTAGTGGAGGAAAAGAAGTTCGGGGCAGAAATAGTACCAGGATTTCAGGCACTATCAGAGGGATGTACCCCTTATGATATCAATCAAATGCTAAATTGTGTGGGAGAACACCAGGCAGCCATGCAAGTCATTAGAGAAATAATCAATGAAGAGGCGGCAGACTGGGACCAGCAACACCCGATACCAGGTCCACTGCCAGCAGGACAACTTAGAGACCCCAGAGGATCAGATATAGCGGGAACCACCAGCACAGTAGAGGAACAAATACAGTGGATGTACAGGGGTCAAAATTCCGTCCCAGTGGGGAACATTTATAGAAGATGGATTCAATTAGGATTGCAGAAATGTGTCAGGATGTACAATCCTACTAATATACTAGATGTAAAACAAGGGCCAAAAGAACCCTTCCAAAGCTATGTAGATAGATTCTACAAAAGCCTACGGGCAGAACAAGCAGACACAGCCGTGAGAGCATGGATGACAGAAACACTACTGGTCCAGAATGCTAACCCAGATTGCAAGCTAGTACTC

>HIV-2(A), GH123

TGTACAACAGACAGGCGGTGGCAACTATATCCACGTGCCACTGAGCCCCCGAACTCTAAATGCTTGGGTAAAATTAGTAGAGGACAAGAAGTTCGGGGCAGAAGTAGTGCCAGGATTTCAAGCACTCTCAGAAGGCTGCACGCCCTATGATATCAACCAAATGCTTAATTGTGTGGGCGATCACCAAGCAGCTATGCAAATAATCAGAGAGATTATCAATGACGAAGCAGCAGATTGGGATGCACAGCACCCAATACCAGGCCCCTTACCAGCAGGGCAGCTTAGAGACCCAAGGGGGTCTGACATAGCAGGAACAACTAGCACAGTAGAAGAACAGATCCAGTGGATGTATAGGCCACAAAATCCCGTGCCGGTAGGGAACATCTACAGAAGATGGATCCAGATAGGGCTACAGAAGTGTGTCAGGATGTACAACCCAACTAACATCTTAGACGTAAAGCAGGGACCAAAGGAACCGTTCCAGAGCTATGTGGACAGGTTCTATAAAAGCTTGAGGGCAGAACAAACAGATCCGGCAGTAAAGAACTGGATGACCCAAACGCTGCTAATACAGAATGCCAACCCAGACTGCAAGTTAGTACTA

>HIV-2(D), L33083

AGTGCAGCAAGTCGGCGGAAATTATGTCCACCTACCGCTGAGTCCCAGAACATTAAATGCATGGGTTAAGTTAGTGGAGGACAAAAAATTCGGGGCAGAGGTAGTGCCAGGGTTTCAGGCACTATCGGAAGGCTGCACTCCGTATGACATCAATCAGATGCTAAATTGTGTAGGAGAACATCAGGCAGCCATGCAGATCATAAGGGAAATAATCAATGATGAGGCAGCAGATTGGGATCAGCAGCATCCACAACCAGGCCCACTACCAGCAGGACAGCTCAGAGATCCACGAGGATCTGATATAGCAGGAACCACTAGCACAGTGGAGGAACAAATACAGTGGATGTACAGGCAGCAGAATCCCATACCAGTTGGAAATATCTATAGGAGATGGATCCAGCTAGGGTTACAGAAATGTGTCAGAATGTACAACCCAACTAACATTCTGGATATAAAACAAGGGCCAAAAGAGACGTTCCAGAGCTATGTAGATAGATTCTACAA

AAGCTTGAGGGCAGAACAAACAGACCCAGCAGTGAAAAATTGGATGACACAAACACTGCTGATTCAGAATGCTAACCCAGATTGCAAGTTAGTACTA

>HIV-2(E), L33087

AGTGCAACAGATAGGAAATAACTATGTGCACTCTCCACTGTCCCCAAGAACATTGAATGCATGGGTCAAATTAGTAGAAGAAAAGAAATTTGGAGCAGAGGTAGTGCCAGGCTTCCAGGCATTATCAGAAGGATGCACCCCGTATGACATCAACCAGATGCTTAATTGCGTGGGGGAACATCAGGCAGCCATGCAAATTATCAGAGAGATAATCAATGAAGAAGCAGCAGATTGGGACGTACAGCATCCAAGAGGGCAACCGCCAGCACAGGGCCTAAGAGACCCATCAGGATCAGACATAGCAGGGACAACCAGTACCCCCGCAGAACAAATAGAGTGGATGTACAGGAATCCAAATCCAATCCCTGTGGGAGACATCTATAGAAGATGGATCCAGCTAGGGCTCCAGAAATGTGTCAGAATGTATAATCCAACAAACATTCTGGACGTCAAACAGGGGCCCAAAGAATCTTTTCAGAGCTATGTAGATAGATTCTACAAAAG

CTTGAGGGCAGAACAAACAGACCCAGCAGTGAAAAATTGGATGACACAAACACTGCTGATTCAGAATGCTAACCCAGATTGCAAGTTAGTACTA

>HIV-2(F), U75441

AGTGCAGCAGGTAGGAGGAAATTACACCCATATTCCTCTGAGTCCGAGGACATTAAATGCTTGGGTTAAATTAGTAGAGGAAAAGAAATTTGGGGCAGAAATAGTGCCAGGCTTCCAAGCATTGTCAGAAGGCTGCACCCCTTATGATATTAATCAAATGTTAAATTGTGTAGGGGAACATCAGGCAGCCATGCAAATAATCAGGGAAATAATCAATGAAGAAGCAGCCGACTGGGATCAGAATCATCCAAGGCAGCTGCCAGCGCCACCAGGGCTGCGTGATCCGTCAGGATCTGACATTGCAGGAACAACTAGTACAGTACAAGAACAGATAGAATGGATGTACAGACAGGGTAACTCAATCCCAGTAGGGGACATTTACAGAAGATGGATCCAAATAGGCCTTCAAAAATGTGTAAGAATGTACAATCCTACTAATATCCTAGATGTAAAACAGGGACCAAAAGAACCATTTCAAAGCTATGTAGATAGATTCTACAAAAG

CTTGAGGGCAGAACAAACAGACCCAGCAGTGAAAAATTGGATGACACAAACACTGCTGATTCAGAATGCTAACCCAGATTGCAAGTTAGTACTA

>HIV-2(H), AY5308

GGTGCAGCAGATAGGTGGCAATTATGCCCACCTACCTCTAAGTCCTAGAACACTCAATGCCTGGGTAAAACTGGTAGAGGAGAAAAAATTTGGAGCAGAAGTAGTGCCAGGATTTCAGGCACTCTCAGAGGGCTGCACGCCCTATGATATTAATCAAATGTTAAATTGCGTGGGAGAACATCAAGCTGCTATGCAAATTATCAGGGAAATAATTAATGATGAAGCAGCAGATTGGGACACACAGCACCCAAACCAAGGCCCACCACCAGCAGGGCAACTTAGAGAGCCAAGAGGTTCTGATATTGCAGGAACAACTAGCACAGTGGAAGAGCAGATACAGTGGATGTACAGGCCGCAAAATCCAATACCGGTGGGTAACATCTATCGGAGATGGATCCAATTGGGCCTACAAAAATGTGTTAGAATGTACAATCCAACTAATATCTTAGATATAAAGCAAGGGCCAAAGGAGCCATTTCAAAGTTATGTAGATAGATTCTACAA

AAGTTTGAGAGCAGAACAAACAGATCCAGCAGTGAAAAATTGGATGACTCAGACGCTGCTGATTCAGAATGCTAACCCAGACTGCAAACTCGTGTTA

>SIVSME041, HM059825

AGTGCAGCAAGTAGGTGGCAATTATACCCACCTACCCTTAAGTCCAAGAACATTAAATGCTTGGGTAAAATTGATAGAAGAGAAAAAATTTGGGGCAGAAGTAGTGCCAGGATTCCAAGCACTATCAGAAGGCTGCACTCCCTATGACATCAATCAGATGCTAAATTGTGTAGGGGAGCATCAATCAGCCATGCAAATTATTAGAGAAATTATAAATGAAGAAGCTGCTGATTGGGATTTACAACACCCACAGCCAGGTCCAATACCAGCAGGACAACTTAGAGACCCGAGAGGATCAGACATTGCAGGAACTACTAGCACAGTAGAAGAACAAATTCAATGGATGTATAGGCAGCAAAACCCTATACCAGTAGGTAACATTTACAGAAGGTGGATCCAATTAGGGCTGCAAAAATGTGTAAGGATGTATAATCCAACAAACATTTTAGATGTGAAACAAGGACCAAAAGAGCCATTTCAAAGCTATGTAGATAGATTCTACAA

GAGTCTAAGAGCAGAACAAACAGACCCAGCAGTGAAAAATTGGATGACTCAAACACTGCTGATTCAAAATGCTAACCCAGATTGCAAATTGGTGCTC

pMD2.G encodes the vesicular stomatitis virus glycoprotein (VSV G) and psPAX2 encodes HIV-1 Gag and Gag-Pol [98]. pCIG3N and pCIG3B encode N-tropic and B-tropic versions of murine leukemia virus (MLV) Gag-Pol and pCNCG is an MLV-derived vector expressing GFP [61,99]. pONY3.1 is an equine infectious anemia virus (EIAV) *gag-pol* plasmid and pONY8.0 is an EIAV GFP-packaging vector [68].

pAPM is a lentiviral vector expressing puromycin-resistance and a miR30-based knockdown cassette from the spleen focus forming virus LTR [48,63,64]. The knockdown targeting sequences used here were as follows: luciferase: 5’-tacaaacgctctcatcgacaag-3’, cyclophilin A (CypA): 5’-ctggattgcagagttaagttta-3’, TRIM5: 5’-tgccaagcatgcctcactgcaa-3’. pAIP and pAIB are lentiviral vectors expressing puromycin and blasticidin resistance respectively. The HIV-1 Env glycoprotein expression plasmid was based on HXB2 [46] and the HIV-2 Env was from the MCN clone [100]. MLV ecotropic Env was expressed from pFBMOSALF [101] and its cognate receptor, mCAT1, was stably expressed with the pBABE-puro MLV-based vector. Codon optimized TvA with a triple HA tag derived from pKZ261 [102] was cloned into pAIP (pAIP-TvA). ALV-A *env* glycoprotein for virion pseudotyping was expressed from pAB6 [103]. Far red fluorescence protein TagRFP-657 [73] was cloned into pAIB for stable expression (pAIB-RFP).

### Cells

Cell lines were either grown in DMEM (293T, TE671, HeLa, NP2, U87, HT1080, and Crandall feline kidney fibroblasts, CRFK cells) or RPMI (Jurkat, SupT1, CEM-SS, Raji, U937, and THP-1), supplemented with 10% fetal calf serum as described before [61,104,105].

PBMC were separated by Ficoll density centrifugation, stimulated with PHA for 3 days, and cultured in RPMI supplemented with antibiotics, 10% fetal bovine serum, and 20 IU/ml hIL-2 [67,106].

CD4^+^ T lymphocytes were enriched from PBMC by positive selection using magnetic beads (Miltenyi Biotec). Typically the resulting population was >99% CD4^+^. Cells were stimulated for 24 hrs on NUNC maxisorp plates that had been coated with 2 μg/ml anti-CD3 antibody and 2 μg/ml anti-CD28 antibody (BD Biosciences) in RPMI with 10% FBS, glutamax (Invitrogen), and 20 IU/ml hIL-2. Two wks after primary stimulation, cells were re-stimulated using plate-bound anti-CD3 and anti-CD28 antibodies.

### Production of viral stocks

VSV G-pseudotyped viral stocks of HIV-1, SIV_MAC_239, and the CA chimera vectors described above, were prepared by co-transfection of the indicated plasmids with pMD2.G in 293T cells, as described [31]. Virion stocks were normalized by reverse transcriptase assay [31] and by titer on non-restrictive CRFK cells or HeLa cells [107]. For production of the shRNA-expressing APM vectors, 8 x 10^6^ cells were plated per 10-cm plate. The next day, cells were transfected using Lipofectamine 2000 (Invitrogen) and 20 μg of pAPM, 15 μg of psPAX2 and 5 μg of pMD2.G. Supernatant was collected and passed through a 0.45 μM filter at 48 hrs and at 72 hrs post-transfection, and used immediately to transduce target cells.

### Challenge with GFP reporter virus

Reporter virus-containing supernatant was titrated onto 4 x 10^4^ of the indicated target cells, in 0.4 ml media per well, in 24-well plates. As_2_O_3_ (Sigma) was prepared as described [31] and, where indicated, added to the cell culture 15 mins prior to virus addition. Cell supernatant was replaced with fresh medium without drug, 12 hrs after addition of virus. Cells were trypsinized when necessary and analyzed by flow cytometry 48 hrs after infection, as described [61].

### RNA interference using lentivirus vectors

Jurkat cells or primary CD4^+^ T cells were spinfected with shRNA-encoding APM vectors twice, at 24 hr and 48 hr after stimulation with plate-bound anti-CD3 and anti-CD28 antibodies. Spinfection was done at 1,130 rcf for 90 mins, using 2 ml of freshly produced virus supernatant for each well of a 6-well plate containing 5 x 10^5^ stimulated lymphocytes. Cells were put in 5 μg/ml of puromycin for 72 hrs, 2 days after the first spinfection.

### Reverse transcriptase assay

Virus-containing supernatant was harvested 48 hr post-transfection, clarified by low-speed centrifugation, and filtered through 0.45 μm pore filters (Sarstedt). Reverse transcriptase (RT) activity in the supernatant was quantified using a modified Sybr green I-based, real-time PCR, enhanced RT assay [108,109]. Virions in cell-free supernatant were disrupted by adding an equal volume of a solution containing 0.25% Triton X-100, 50 mM KCl, 100 mM Tris-HCl pH 7.4, and 0.4 U/μl RNase inhibitor (RiboLock, MBI Fermentas). Virion lysate was then added to a single-step, RT PCR assay with 35 nM MS2 RNA (Roche) as template, 500 nM of each primer (5’-TCCTGCTCAACTTCCTGTCGAG-3’ and 5’-CACAGGTCAAACCTCCTAGGAATG-3’), and hot-start Taq (Promega), all in 20 mM Tris-Cl pH 8.3, 5 mM (NH_4_)_2_SO_4_, 20 mM KCl, 5 mM MgCl_2_, 0.1 mg/ml BSA, 1/20,000 SYBR Green I (Sigma), and 200 μM dNTPs. All reactions and quantitation of product were carried out with a Biorad CFX96 cycler. The RT step was 42°C for 20 min, and the PCR was programmed for 40 cycles of denaturation at 95°C for 5 s, annealing 55°C for 5 s, extension at 72°C for 20 s and acquisition at 80°C for 5 s. A standard curve was obtained using known concentrations of recombinant HIV-1 RT (Ambion).

### Quantitation of viral cDNA

Cell-free virions were normalized by RT-activity and incubated with CRFK, Hela or Jurkat cells in 6-well plates for 12 hrs, for full-length linear cDNA and 2-LTR circles, or 48 hrs, for Alu PCR. For each virus and cell type, infections were also performed in the presence of 40 μM AZT, to control for contamination of plasmid DNA in the PCR reaction. Cells were harvested and washed extensively with PBS. Total DNA was extracted (Qiagen, Qiamp DNA mini kit), quantified, and subjected to real-time PCR with a Biorad CFX96 cycler.

Full-length linear retroviral cDNA and 2-LTR circles were detected with SYBR-Green I based reactions using 100 ng template DNA and 320 nM of each primer pair (5’-ACAAGCTAGTACCAGTTGAGCCAGATAAG-3’ and 5’-gccgtgcgcgcttcagcaagc-3’ for full length linear; 5’-AACTAGGGAACCCACTGCTTAAG-3’ and 5’-TCCACAGATCAAGGATATCTTGTC-5’ or 5’- CAGTGTGGAAAATCTCTAGCAGTAC-3’ for 2-LTR circles) in 20 mM Tris-Cl pH 8.3, 5 mM (NH_4_)_2_SO_4_, 20 mM KCl, 5 mM MgCl_2_, 0.1 mg/ml BSA, 1/20,000 SYBR Green I (Sigma), and 200 μM dNTPs. The PCR was programmed for 40 cycles of denaturation at 95°C for 5 s, annealing 55°C for 5 s, extension at 72°C for 20 s and acquisition at 80°C for 5 s. Provirus was quantified by Taqman-based ALU-PCR according to the protocol described by Butler et al. [69] using 200 ng of template DNA, primers 5’-AACTAGGGAACCCACTGCTTAAG-3′ and 5′-TGCTGGGATTACAGGCGTGAG-3′ and probe 5′-(FAM)-ACACTACTTGAAGCACTCAAGGCAAGCTTT-(TAMRA)-3′. PCR was performed with a CFX96 cycler (Biorad): 95°C for 15 seconds and 60°C for 90 seconds, for 50 cycles. Relative quantification of retroviral cDNA sequences and ALU PCR was with respect to standard curves prepared from serial dilutions of DNA derived from the cell culture with the highest infection, diluted in DNA extracted from non-infected cells.

### Microscopy

CRFK and Jurkat cells transduced with VSV G-pseudotyped HIV-1_NL4-3_GFP or SIV_MAC_239-GFP vectors were visualized by phase contrast and fluorescence microscopy 4 days after vector challenge. Pictures of live cell cultures were taken at 100x magnification using a Nikon Eclipse Ti microscope equipped with a DS-QiMC digital camera and NIS elements software.

### Heterokaryon assay

2 x 10^7^ Hela-RFP and 2 x 10^7^ Jurkat-TvA were washed with serum-free DMEM and slowly resuspended over 1 min in 500 μl of Polyethylene Glycol 1500 (PEG-1500, GE Healthcare), at 37°C. Cells were incubated for another 2 mins and then 2 ml of serum-free DMEM was added slowly over a period of 4 minutes at 37°C with constant, gentle agitation. An additional 5 ml of serum-free DMEM was added and cells were incubated for 5 min at 37°C. Cells were then pelleted and resuspended in complete medium before seeding in 24-well plates. 6 hours later, cells were challenged with ALV-A Env-pseudotyped vectors. A negative fusion control sample was also produced with no PEG addition. Infected cell cultures were analyzed using a FACS-Canto (BD) 48 hrs after vector challenge. Fluorescence acquisition was performed using blue (488 nm) and red (633 nm) lasers. Dead cells were excluded from the analysis based on propidium iodide staining.

### Ethics statement

Human peripheral blood mononuclear cells (PBMC) were obtained from anonymous, untraceable blood donors. This research is therefore considered non-human subjects research by our Institutional Review Board, based on NIH guidelines (45 CFR 46.102(f)): http://grants.nih.gov/grants/policy/hs/faqs_aps_definitions.htm.
